# Charting Regions of Cobalt’s Chemical Space with Maximally Large Magnetic Anisotropy: A Computational High-Throughput Study

**DOI:** 10.1021/jacs.4c14076

**Published:** 2024-11-27

**Authors:** Lorenzo A. Mariano, Vu Ha Anh Nguyen, Valerio Briganti, Alessandro Lunghi

**Affiliations:** School of Physics, AMBER and CRANN Institute, Trinity College, Dublin 2, Ireland

## Abstract

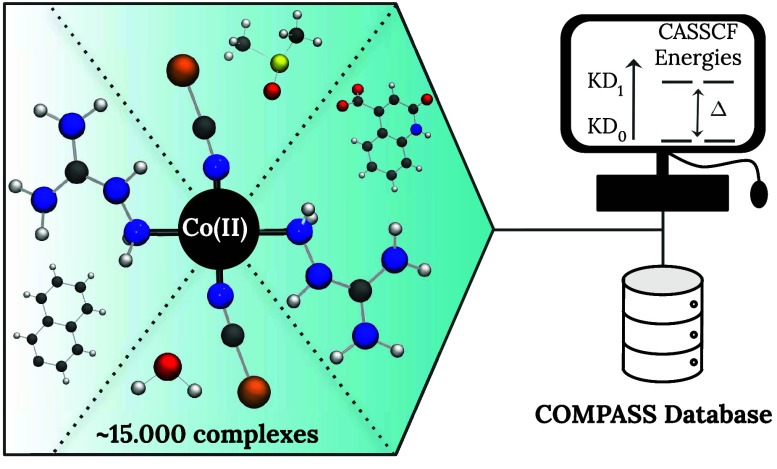

Magnetic anisotropy slows down magnetic relaxation and plays a prominent role in the design of permanent magnets. Coordination compounds of Co(II) in particular exhibit large magnetic anisotropy in the presence of low-coordination environments and have been used as single-molecule magnet prototypes. However, only a limited sampling of cobalt’s vast chemical space has been performed, potentially obscuring alternative chemical routes toward large magnetic anisotropy. Here we perform a computational high-throughput exploration of Co(II)’s chemical space in search of new single-molecule magnets. We automatically assemble a diverse set of ∼15,000 novel complexes of Co(II) and fully characterize them with multireference ab initio methods. More than 100 compounds exhibit magnetic anisotropy comparable to or larger than leading known compounds. The analysis of these results shows that compounds with record-breaking magnetic anisotropy can also be achieved with coordination four or higher, going beyond the established paradigm of two-coordinated linear complexes.

## Introduction

Magnetic anisotropy describes how the energy of a magnetic moment varies with its orientation in space and is at the origin of slow magnetic relaxation^1^ and permanent magnetism.^2^ At the microscopic level, magnetic anisotropy arises from the interaction between the spin, *S*, and orbital motion, *L*, of an ion’s unpaired electrons, known as spin–orbit coupling *Ĥ*_SOC_ = λ*L*·*S*, where λ is the many-electrons spin–orbit coupling constant. Once the symmetry of the space surrounding the ion is broken by embedding it in a solid-state lattice, spin–orbit coupling establishes a preferential direction in space for the ion’s magnetic moment. Among the chemical compounds exhibiting this property, cobalt represents the prime example among transition metals and its large magnetic anisotropy has been reported for materials ranging from elemental Co^3^ and intermetallic alloys^4^ to molecules^5^ and surface-adsorbed atoms.^6^

In the case of molecules and atoms in particular, record values of anisotropy have been observed.^5,6^ Such groundbreaking results had been achieved by maximizing the value of *L*, which correlates with spin–orbit coupling strength. While most coordination geometries lead to a quench of an ion’s orbital angular momentum, low coordination such as two-coordinated linear Co(II) ions can maintain the maximally allowed value of *L* = 3 and therefore exhibit large magnetic anisotropy.^5^ Despite these extensive research efforts, this design rule poses serious challenges. Indeed, synthesizing linear two-coordinate Co compounds is quite challenging and only four compounds have been reported to date.^5,7^ Moreover, low coordination environments for cobalt ions lead to very reactive compounds limiting the scope of these molecules outside the confines of fundamental science. Alternatively, higher coordination numbers have also been explored, predominantly coordination 4, but achieving large angular momentum *L* has proved to be hard as it requires promoting low-lying excited electronic states through large distortions from the ideal geometry with *T*_d_ symmetry.^8^ Although there is ample scope for the exploration of other chemical strategies for the realization of highly anisotropic cobalt-based molecules, the intrinsic rarity of magnetism^9^ and the vastness of the chemical space hamper further progress.^10^

Computational approaches and large-scale ab initio screening frameworks in particular are becoming leading tools to accelerate the discovery of new materials with desired properties.^11−14^ Seminal attempts to use such high-throughput approaches in the field of magnetism have targeted the determination of the spin ground state of spin crossover compounds^15^ and solid-state Heusler-type magnets.^16^ However, these studies are invariably performed with density functional theory (DFT), which is known to lead to qualitatively wrong predictions of magnetic anisotropy in coordination compounds.^17^ On the other hand, attempts to systematically explore different coordination geometries for Co(II) monometallic coordination compounds with accurate multireference ab initio methods have been confined to model systems with a single ligand or a handful of structures from crystallography databases.^18−22^ Despite being very informative for the molecules at hand, this approach cannot account for the vast chemical and structural diversity of organic ligands’ chemical space.

In this study, we remove the main roadblock to the systematic simulation of magnetic anisotropy by establishing a multireference ab initio high-throughput framework. Our study addresses around 15,000 Co(II) monometallic coordination compounds and individuates tens of candidates with record-large magnetic anisotropy. Most importantly, these record values are also achieved with coordination numbers higher than two and unprecedented coordination geometries. A new general design rule emerges from data with the potential of extending the scope of chemical synthesis in achieving molecules with simultaneous large magnetic anisotropy and high chemical stability.

## Results

### High-Throughput Ab Initio Screening

This section presents the computational strategy used to construct and analyze the target complexes in this study. The results have been compiled into an open-access database named CObalt-based Magnetic Properties from Ab initio Structural Study (COMPASS). Details about the information contained in the COMPASS database are discussed below, and access to COMPASS is explained in the Data Availability section.

**Figure 1:**
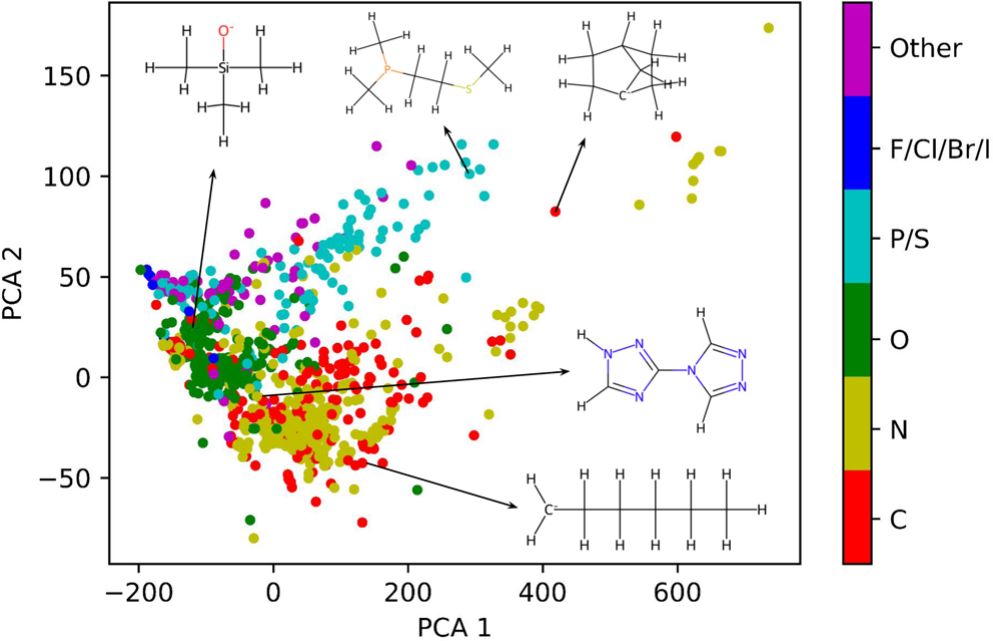
Principal component analysis (PCA) for the whole set of monodentate ligands with less than 20 atoms. Different colors are associated with the different nature of the bonding atom as reported in the colorbar. The representation of a random selection of ligands is also reported.

The first step of our computational strategy is collecting all molecules containing cobalt from the Cambridge crystallographic structural database (CCSD).^23^ We then select all crystals that only contain co-based monometallic coordination compounds and extract the associated organic ligands. The latter are then classified by the number of binding atoms, leading to a set of 1423 monodentate ligands deposited in COMPASS_lig2 database. The total number of coordination of compounds that can be generated with such a set of ligands is virtually infinite due to the combinatorial nature of the problem and constraints on the coordination motifs are introduced to make a systematic computational study possible. Figure 1 reports the results of a principal component analysis (PCA). This numerical technique is widely used to simplify the representation of high-dimensional data. In the present case, PCA is applied to the bispectrum components of the ligand’s binding atom, which are a mathematical representation of the 3D chemical environment of the atom. Each point of Figure 1 therefore represents a ligand and the distance among points is taken as a measure of their chemical and structural similarity. Based on this and capping the maximum size of the ligands to 20 atoms, the furthest point sampling (FPS) method is used to select a subset of ligands with maximal chemical diversity, leading to a final set of 208 ligands that well represent the coordination chemistry of Co as available in CCSD. These ligands are deposited in COMPASS_lig1 database.

Cobalt compounds with the largest magnetic anisotropy to date are found among linear two-coordinated and distorted tetrahedral ones. Based on this, we use the software MolSimplify^24^ to assemble a comprehensive data set of tetrahedral Co(II)-based compounds with general formula CoA_2_B_2_, and linear two-coordinated compounds of the form CoA_2_. Only two different ligands at the time are allowed for the tetrahedral geometry with the 2-fold objective of making the final compounds more likely to be successfully synthesized and constraining the total number of compounds to be simulated. The ligands A and B are systematically chosen from our COMPASS_lig1 data set, leading to 21,528 4-coordinate compounds and 208 2-coordinate compounds.

The total molecular charge is set based on the ligands’ charges, determined with the help of DFT, and considering cobalt in its 2+ oxidation state. All compounds were considered in their high-spin configuration, known for exhibiting large magnetic anisotropy in the chosen crystal field geometries.^25^ The structure of each compound is optimized without imposing any constraint on the molecular symmetry. The final geometries and molecular orbitals are then used to run a multireference calculation and compute the spin Hamiltonian parameters describing the magnetic properties of the entire set (vide infra).

The total number of compounds which survive both geometry optimization and multireference calculation is 15,559 for coordination number 4 and 49 for coordination number 2. The lower success rate for coordination 2 is due to the high instability of this low coordination number, often resulting in unacceptable optimized geometries. In the following, we will refer to the set obtained by combining 4 ligands as **Set-1** and the set obtained by combining 2 ligands as **Set-2**. The former constitutes our COMPASS_set1 database, while the latter is stored in the COMPASS_set2 database.

### Magnetic Anisotropy

Co(II) ions possess seven electrons in their d shell, leading to a high spin ground state with *S* = 3/2. The 2*S* + 1 = 4 states of such a spin, split in energy by the presence of a crystal field and spin–orbit coupling, can be described by the conventional spin Hamiltonian
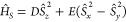
1where *D* and *E* are the axial and rhombic zero-field splitting parameters, respectively. Magnetic anisotropy is defined as the difference in energy between the first and second Kramers doublets. For vanishing values of *E*, these states correspond to the maximal and minimal projections of the spin along the *z* axis, *M_S_* = ±3/2 and *M_S_* = ±1/2, respectively, and their energy difference is 2*D*. In the presence of negative values of the parameter *D*, the energy of the maximally large projections of the spin along the *z* axis (*M_S_* = ±3/2) is stabilized, leading to an easy-axis magnetic anisotropy, known to be favorable for single-molecule magnets.^27,28^ Positive values of *D* correspond to easy-plane magnetic anisotropy. The parameter *E* leads to the mixing of different values of *M_S_*, promoting quantum tunneling of the magnetization and undercutting the benefits of large *D* values.^29^ The requirement of maximizing |*D*/*E*| is therefore key to designing single-molecule magnets.

Electronic properties and spin Hamiltonian parameters were computed using multireference CASSCF method with an active space containing seven electrons in the five 3d orbitals of the cobalt. State average procedure has been performed over 10 quartets and 40 doublets. Spin–orbit coupling was treated using quasi-degenerate perturbation theory. Further details about the ab initio calculations are provided in the Methods section. All compounds were found to have a quartet ground state. The distance from the first doublet is reported in Figure S4 of the Supporting Information.

**Figure 2:**
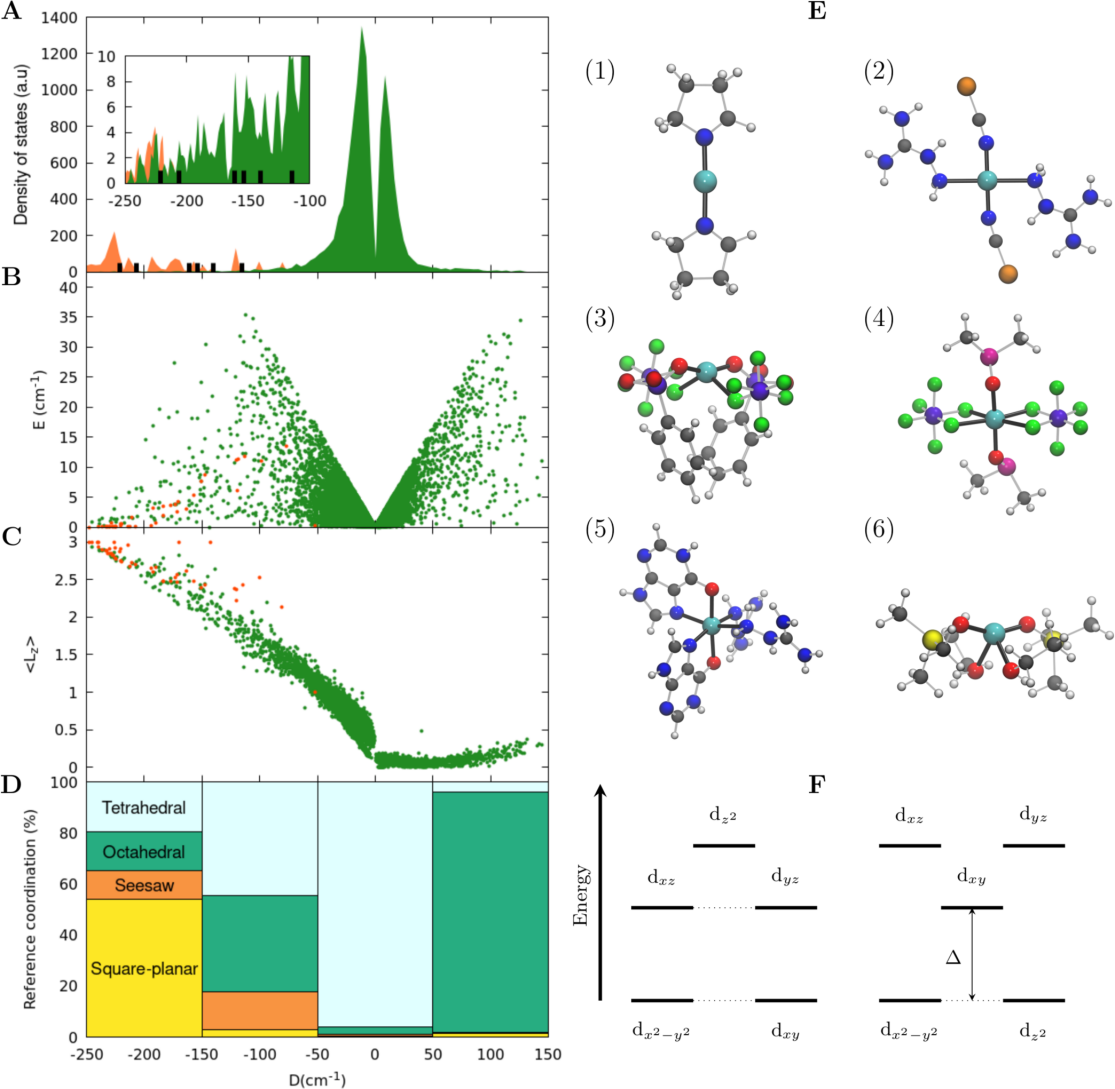
(A): Distribution of the computed anisotropy *D* values for **Set-1** (green) and **Set-2** (orange). The density of states for **Set-2** is multiplied by 50 to facilitate the visualization. Black bars correspond to literature values of [Co(II)(C(SiMe_2_ONaph)_3_)_2_] (Me = methyl, Naph = naphthyl),^5^ [Co(II)(sIPr)NDmp] (Dmp = 2,6-dimesitylphenyl),^7^ (Ph_4_P)_2_[Co(II)(C_3_S_5_)_2_],^26^[Co(II)(IPr)NDmp] (Dmp = 2,6-dimesitylphenyl),^7^ [Co(II)(cyIPr)NDmp] (Dmp = 2,6-dimesitylphenyl),^7^ (HNEt_3_)_2_[Co(II)(L)_2_] (H_2_L = 1,2-bis(methanesulfonamido)benzene),^8^ from the most negative to the least negative, respectively. In the inset, only values of *D* < −100 cm^–1^ are reported and no multiplication factor has been applied for **Set-2.** (B): Computed values of rhombic parameter *E* with respect to anisotropy *D*. Green dots is used for **Set-1** and orange dots for **Set-2.** (C): Expectation value of the *z*-component of the orbital angular momentum *L* for the ground-state Kramers doublet as a function of the computed anisotropy *D*. Values are reported for **Set-1** (green) and **Set-2** (orange). (D): The relative percentage of each reference polyhedron in **Set-1** across different *D* ranges is represented as follows: yellow for square-planar, orange for seesaw, sea green for octahedral, and light cyan for tetrahedral. (E): Representative geometries for different reference coordination in the two sets. (1) linear, (2) square planar, (3) seesaw, (4) octahedral with large negative *D*, (5) octahedral with large positive *D*, (6) tetrahedral,. Color code: cyan for Co, blue for N, red for O, gray for C, white for H, orange for Se, yellow for Si, green for F, magenta for S, and purple for P. **F**: molecular orbital diagram for linear coordination (left) and tetrahedral coordination (right).

The computed values of *D* are reported in Figure 2A. For **Set-1**, the distribution of values of *D* shows two pronounced peaks around *D* ∼ 0 cm^–1^, indicating that a random selection of ligands is likely to result in complexes with small nonzero magnetic anisotropy. In contrast, for **Set-2**, the majority of compounds exhibit large negative *D* values and no positive values. The large size of **Set-1** allows to sample the tails of the distribution which are found to extend to significantly large values of |*D*|. The inset of Figure 2A reveals 196 complexes from **Set-1** and 47 from **Set-2** with magnetic anisotropy *D* < −100 cm^–1^, which is comparable to the best compounds synthesized to date.^5,7,8,26^ Further exploration in the region of low *D* values reveals 22 compounds from **Set-1** and 27 compounds from set **Set-2** with magnetic anisotropy smaller than −200 cm^–1^, comparable to the record values of this property for cobalt.^5,7^ Interestingly, a significant number of compounds with large positive values of *D* have been detected in **Set-1**. Figure 2A also reports the value of *D* for the six complexes with the highest negative magnetic anisotropy reported in the literature. Four of these compounds exhibit linear coordination of the central Co(II) with *D* values of −221,^5^ −206,^7^ −154,^7^ and −140 cm^–1^.^7^ The other two compounds instead feature distorted tetrahedral coordination and *D* ∼ −113^8^ and −161 cm^–1^.^26^ The values of the rhombic parameter *E* are presented in Figure 2B, where the theoretical limit |*E*/*D*| < 1/3 is clearly visible. Interestingly, the parameter *E* spans a large range of values in both **Set-1** and **Set-2** except for *D* < −200 cm^–1^, suggesting that maximizing *D*/*E* is possible.

### Angular Momentum, Coordination Geometries, and Molecular Orbitals

To test the hypothesis that large magnetic anisotropy emerges from unquenched angular momentum,^5,30−32^ we calculated the expectation value of the operator *L_z_* for the ground-state Kramers doublet for each compound. These results are presented in Figure 2B. A consistent correlation between magnetic anisotropy and ⟨*L_z_*⟩ is observed across all compounds, reaching the maximum theoretical value of 3 for the most anisotropic ones. In light of the observed relationship between ⟨*L_z_*⟩ and magnetic anisotropy, we now examine the geometric arrangement of atoms surrounding the magnetic ion, as well as the resulting energy distribution and occupation of the 3d molecular orbitals (MOs). In the following, we first focus on **Set-2**, followed by the analysis of the compounds in **Set-1**.

The vast majority of compounds prepared in linear coordination maintain the angle θ between the Co ion and the 2 coordinated ligands close to 180° (see Figure 2E-(1)). It is possible to understand why this geometry supports large values of ⟨*L̂_z_*⟩ by considering the MO diagram for a linear compound, as shown in the left panel of Figure 2F. The 3d electronic states are ordered as *E*(d_*z*^2^_) > *E*(d*_xz_*, d*_yz_*) > *E*(d_*x*^2^–*y*^2^_, d*_xy_*). The degeneracy of the d_*x*^2^–*y*^2^_ and d_*xy*_ orbitals (*m*_*l*_ = ± 2), and the d*_xz_* and d*_yz_* orbitals (*m*_*l*_ = ± 1), leads to unquenched orbital angular momentum if there is an odd number of electrons in one of these two manifolds. Following the Aufbau principle to populate the 3d orbitals, the resulting occupation would be (d_*z*^2^_)^1^(d*_xz_*, d*_yz_*)^2^(d_*x*^2^–*y*^2^_, d*_xy_*)^4^, leading to a final ⟨*L_z_*⟩ = 0. However, a non-Aufbau filling of 3d orbitals would result in the configuration (d_*z*^2^_)^1^(d*_xz_*, d*_yz_*)^3^(d_*x*^2^–*y*^2^_, d*_xy_*)^3^, yielding ⟨*L_z_*⟩ = 3. In the set of compounds studied here, large values of ⟨*L_z_*⟩ are indeed found for nearly linear geometries, leading to the conclusion that the large magnetic anisotropy for these compounds is due to the non-Aufbau occupation of the 3d orbitals. A closer inspection of the trend of θ (reported in Figure S1), reveals that deviations from linearity correlate with a decrease in the computed *D* and ⟨*L_z_*⟩ values. This decrease is explained by a loss of degeneracy between the pairs of orbitals d_*x*^2^–*y*^2^_ – d*_xy_* and d*_xz_*– d*_yz_*.

Interestingly, large values of ⟨*L_z_*⟩ and *D* surpassing those reported in the literature have been detected in compounds with coordination higher than two in **Set-1**. Despite the initial complexes being prepared in tetrahedral coordination, DFT optimization leads to equilibrium geometries that significantly deviate from it. In particular, we note that coordination larger than 4 is also established because some ligands possess a second donor atom despite being singly coordinated in the originally deposited crystallographic structure. In some cases, DFT geometrical optimization then leads to compounds with coordination 6, where two ligands act as bidentate.

The first coordination shell geometry of each compound in **Set-1** is interpreted in terms of ideal polyhedrons as detailed in the Methods section.^33,34^ In the final optimized geometries of **Set-1**, compounds close to square planar, tetrahedral, seesaw, and octahedral are present and Figure 2D reports the relative percentage of each reference polyhedron in different windows of *D* values. In particular, we observe that in the range of *D* values smaller than −150 cm^–1^, the majority of the complexes relax to geometries close to square planar, seesaw or octahedral coordination (see Figure 2E-(2–4)). Further inspection of these compounds reveals the establishment of two sets of metal-to-ligand distances in the same compounds, with two ligands lying along the same direction in a pseudolinear fashion and exhibiting short bond lengths and the remaining ligands lying in a perpendicular plane and showing longer bond lengths. These geometries lead to strong axial anisotropy and the emergence of pseudolinear MO configuration, as schematically reported in Figure S2. In contrast with the case of linear two-coordinated compounds, the degeneracy of the d*_xz_*, d*_yz_* and d_*x*^2^–*y*^2^_, d*_xy_* orbitals is partially lifted by the presence of the equatorial ligands. Nevertheless, the weak interaction between the metal and the equatorial ligands results in a small energy splitting between these orbitals such that unquenched orbital angular momentum is still supported. Interestingly, when the four equatorial donor atoms of octahedral geometries lie close to the metal and the metal-axial bond length is elongated (see Figure 2E-(5)), significant in-plane anisotropy is observed. Finally, tetrahedral coordination remains favorable for compounds with magnetic anisotropy values ranging from −150 and 50 cm^–1^. In this case, large magnetic anisotropy is associated with a small energy gap between the d_*x*^2^–*y*^2^_ and d*_xy_* orbitals.^8^ As illustrated in the right panel of Figure 2F, as the separation Δ between *E*(d*_xy_*) and *E*(d_*x*^2^–*y*^2^_) vanishes, ⟨*L_z_*⟩ approaches the value of two and magnetic anisotropy becomes large. Computed values of Δ are reported in Figure S3.

Interestingly, the presence of unquenched orbital angular momentum suggests that the effective spin Hamiltonian of eq 1 might not be the ideal representation of the electronic structure of these molecules. However, we remark that here we are only interested in individuating compounds with large uniaxial anisotropy, which are conveniently described by the sign and magnitude of the coefficient *D*. Moreover, we observe a very good mapping between the coefficient *D* and the ab initio energies, suggesting that the spin Hamiltonian remains at least qualitatively valid for all our compounds. Therefore, although eq 1 might need to be substituted with a better model for studying finer aspects of the spin dynamics, it does not affect the conclusions of the present study.

### Coordination Sphere Analysis

Most of the ligands in the initial set bind to Co with nitrogen (32.7%), carbon (27.9%), and oxygen (21.6%). This distribution is reflected in the composition of the first-coordination sphere of complexes from **Set-1**, where 8694 contain nitrogen, 7384 contain carbon, and 6415 contain oxygen. Interestingly, when the analysis is repeated considering only compounds with computed *D* values smaller than −100 cm^–1^, the statistic is reversed, with most of the 197 compounds containing oxygen (146), followed by nitrogen (98) and carbon (24). To understand the role of oxygen in maximizing magnetic anisotropy, we need to consider the MO diagram in Figure 2F. Such a configuration can be obtained in the case of strong π-donor ligands in the axial direction and weak bonding ligands in the equatorial plane. This situation ensures an energy destabilization of the nonbonding d*_xz_*, d_*yz*_ orbitals with respect to the d_*x*^2^–*y*^2^_, d*_xy_* orbitals. Highly electronegative elements, such as oxygen, are optimal donor atoms to enhance the metal–ligand π interaction because of the presence of multiple lone electron pairs that can establish chemical bonds with the magnetic ion. Ligands of this type have been found in around 65% of the compounds with magnetic anisotropy smaller than −200 cm^–1^. Oxygen, carbon and nitrogen are all present in the first coordination shell of compounds in **Set-2**, but the small size of the latter does not allow the extraction of statistically significant information about their relative importance.

## Discussion

The community of molecular magnetism has been actively pursuing the synthesis of Co single-molecule magnets for over two decades and many different chemical strategies have been successfully pursued.^25^ Very often these efforts are driven by assuming the existence of a direct mapping between coordination geometry and magnetic properties. Our results show that while coordination geometry indeed plays a key role in shaping the magnetic anisotropy of an ion, it cannot be separated by other key factors such as the cross-correlations among the ligands’ different ability to form coordination bonds. Thanks to an unprecedented large-scale screening of Co compounds, we have here uncovered several examples where prominent values of zero-field splitting are achieved in unexpected coordination geometries, ranging from square planar and seesaw to octahedral.

**Figure 3:**
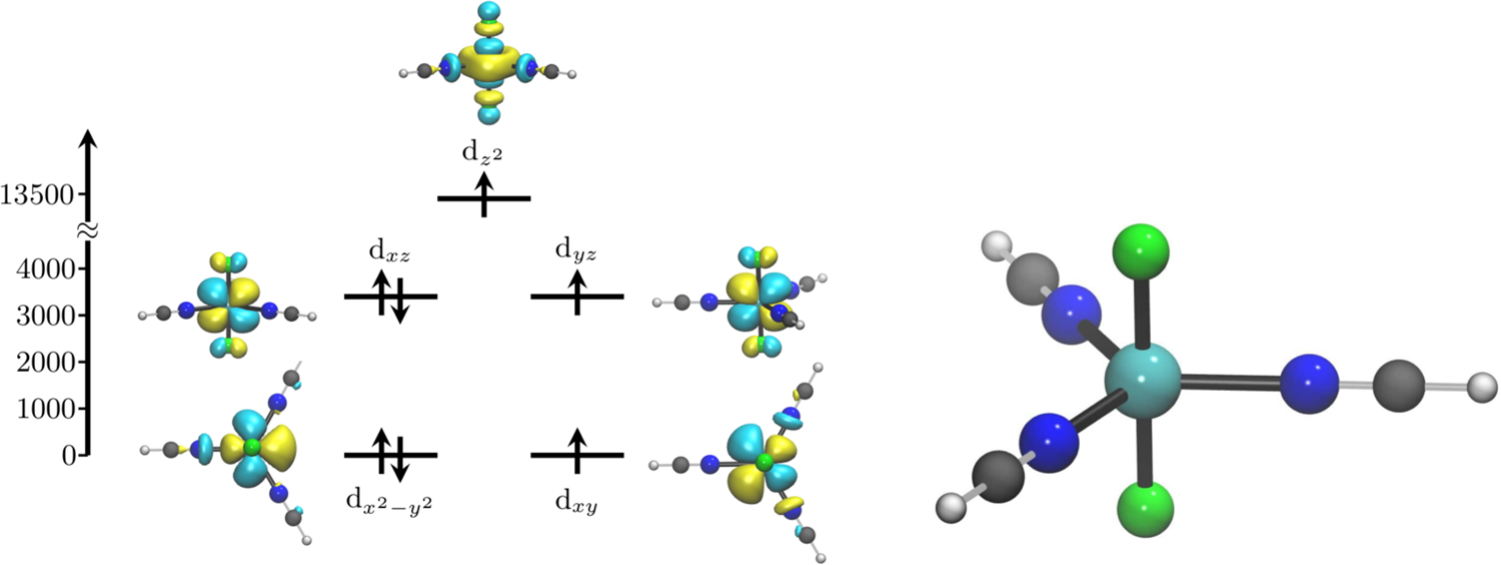
MOs diagram, orbitals occupation, and optimized DFT geometry for the proposed trigonal-bipyramidal compound. CASSCF orbitals are shown next to each 3d electronic level. Color code: cyan for Co, blue for N, gray for C, white for H, and green for F.

We anticipate that the synthesis of the compounds reported here will come with significant challenges, as is common for computational design studies of this kind.^35−37^ In particular, it is currently beyond the state of the art to systematically explore the chemical stability and synthetic feasibility of coordination compounds against the immense number of chemical environments, possible crystal structures and competitive reactions that can take place in real experiments. Nonetheless, a very clear and actionable design rule emerges from our study: a maximal value of angular momentum, and therefore of axial magnetic anisotropy, can be achieved with pseudolinear geometries, where two strong donors are aligned along the same direction and only weak donors are present in the equatorial plane. To further support the universality of our claims, we assemble a complex with trigonal pyramidal geometry where axial and equatorial ligands are assigned based on our design rule. Figure 3 reports the optimized structure and MOs for a complex with two fluorine ions placed on the main symmetry axis and three NCH ligands on the equatorial positions. As expected from the position of these ligands on the spectrochemical series, the geometry shows a distance of 1.84 Å between the apical F^–^ and the Co(II) ion, while the distance between the NCH ligands and the metal is 2.14 Å. Complete intramanifold degeneracy is achieved for (d*_xz_*, d*_yz_*) and (d_*x*^2^–*y*^2^_, d*_xy_*), respectively, thanks to the *C*_3_ symmetry axis. The result is a computed magnetic anisotropy of 253 cm^–1^, the largest theoretical value reported so far. The anisotropy of Co in such coordination geometry had been studied experimentally before, but only marginal values of *D* had been achieved due to the lack of the specific tuning of the ligands’ reciprocal donor strength that emerged from this study.^38−40^ Although linear two-coordinate compounds statistically exhibit a larger magnetic anisotropy than any other coordination, compounds with higher coordination numbers have the potential to lead to molecules with improved chemical stability, a necessary condition for any application of these compounds. A similar strategy has recently emerged in the field of air-stable lanthanide single-molecule magnets,^41−43^ supporting the present analysis.

Despite its large-scale nature, the present study is far from a comprehensive exploration of the entire chemical space of these compounds and further efforts in this direction are advisible. While for the time being high-throughput methods will still be necessary, the design of more efficient strategies for the exploration of the chemical space of magnetic molecules will be necessary for multiple reasons: (i) to improve the chances of identifying compounds with intrinsically rare properties, and (ii) reduce the impact of simulations on energy consumption and green-house emissions^44,45^ (an estimate of these is provided in the Supporting Information, Table S2). We argue that this study is an essential step in this direction. For instance, we anticipate that the associated data set COMPASS will serve as a starting point for the generation of effective models able to predict magnetic anisotropy at a much reduced computational cost than ab initio methods and further propel the exploration of the chemical space of these coordination compounds. Machine-learning methods hold a special place in this area^13^ and some early development of models able to predict molecular magnetic anisotropy have already appeared in the literature.^46−50^ However, none of these models has yet been tested against a challenging, diverse, and realistic training set as the present one and we anticipate that this will become a critical benchmark for machine learning.

In conclusion, we have developed a high-throughput multireference ab initio framework to automatically assemble over 15,000 coordination compounds of Co(II) and compute their magnetic anisotropy. We discovered tens of molecules with record values of magnetic anisotropy and a general new chemical design rule that does not invoke low coordination numbers. We expect that these results will facilitate further large-scale explorations of magnetic molecules’ chemical space as well as the experimental characterization of a new class of cobalt compounds with unprecedented properties.

## Methods

### Ligands Database Generation

A total of 47,427 structures containing Co were downloaded from CSD on April 2023. Cif files presenting structural disorder are discarded. The remaining cif files are then converted to *xyz* with the software Atomsk.^51^ The *xyz* files are further processed with the tool cif2xyz, available as part of the software MolForge, available at https://github.com/LunghiGroup/MolForge/. The tool FindMols, also available in MolForge, is then used to individuate molecular entities inside the unit cells and to remap them across the periodic boundary conditions. Crystals containing a unique monometallic molecule of Co are retained, leaving 35,632 entries. For each molecule, the ligands are identified with the tool FindMols. The general formulas of ligands are compared to remove duplicates. We further differentiate ligands by the number of donor atoms and select the monodentate ones. This leaves us with a list of 1424 unique ligands. Bispectrum components are used to represent the structure of the donor atom’s chemical environment in each ligand. Bispectrum components^52^ are built with a cut off distance of 4.1 Å and order 2*J* = 8. PCA is then performed on bispectrum components to obtain a 2D representation of the structural similarity of ligands’ donor atoms. Then, FPS was used to select 208 points on this map that best represented the space, i.e., 208 ligands that are most chemically diverse out of the initial list of ligands. The charge of each ligand is determined using DFT with the same functional and basis set employed for the geometrical optimization (see below). Without performing geometrical optimization, single-point calculations are conducted by varying the total charge of the compound from 2– to 2+ and adjusting the spin multiplicity accordingly. The charge is determined based on the state with the lowest total energy. No open-shell solutions are found to be the most stable configuration for the analyzed ligands. In the case of carbon donor atoms, carbanion solutions are selected by default.

### Generation of Co(II) Compounds

Starting from the geometries of the selected 208 ligands, along with their associated charges and docking atoms, the software molSimplify^24^ is used to generate the initial geometries of the CoA_2_B_2_ (**Set-1**) and CoA_2_ (**Set-2**) compounds by exploring all possible combinations of the ligands within each set. The compounds with a general formula CoA_4_ are not considered as they are not expected to lead to significant symmetry breaking to support large magnetic anisotropy. For **Set-1**, we use the template for tetrahedral coordination, while for **Set-2**, the linear coordination template is applied. Aside from the steric repulsion minimization performed by the molSimplify routine, no additional force-field options are used.

Cobalt is considered to be in its high spin configuration (*S* = 3/2) in all subsequent calculations. Preoptimization of the generated geometries is performed only for compounds in **Set-1** using semiempirical tight-binding DFT (TB-DFT) with the GFN2-xTB method,^53,54^ as implemented in the ORCA 5^55^ software. The optimization threshold is set to 10^–6^ Ha (tight level). Structures for which TB-DFT optimization fails to converge after 100 cycles are replaced with the initial structures generated using molSimplify. Finally, all the structures from both sets are optimized at the DFT level using ORCA 5.^55^ We employ the BP86 functional^56,57^ with the addition of dispersion correction at the D3-BJ level.^58^ All parameters for dispersion corrections are kept at their default values. The def2-TZVPP basis set is used for all atoms. Calculations that do not converge after 500 geometry optimization cycles are discarded. At the end of the optimization run, unrestricted natural orbitals (UNO) are generated for the subsequent multireference calculations.

### Multireference Calculations

Multireference calculations are performed using the State-Average Complete Active Space Self Consistent Field (CASSCF) theory as implemented in the software ORCA 5.^55^ The active space used to build the CASSCF wave function is (7,5), i.e., seven electrons in five 3d-orbitals. The right active space is automatically selected using the Löewdin orbital composition to assess the percentage of Co d orbitals in each molecular orbital (MO). If any of the last five occupied orbitals have less than 30% d character, the highest-energy occupied orbitals (outside these last five) with more than 30% d character are identified and substituted into the active space. The CASSCF calculation is then run with these swapped orbitals. This procedure is initially applied to the UNO orbitals from DFT and then to each subsequent CASSCF run until the active space has the correct composition. The state average procedure is performed using 10 quartet states (2*S* + 1 = 4) and 40 doublet states (2*S* + 1 = 2). Mean-field spin–orbit coupling operator, along with quasi degenerate perturbation theory (QDPT), is employed to account for the mixing of spin-free states. The spin Hamiltonian reported in eq 1 is built using the lowest two Kramers doublets. The Douglas–Kroll–Hess (DKH) scalar relativistic correction is applied to the electronic Hamiltonian, with picture change effects considered up to second order to include DKH corrections in the spin–orbit coupling operator. We use the DKH-def2-TZVPP basis set for all atoms, except for Sn, for which the SARC-DKH-TZVPP basis set is employed. The Δ values reported in Figure 2F were extracted from the eigenvalues of the ligand field one-electron matrix built using Ab Initio Ligand Field Theory (AILFT) on top of the CASSCF orbitals.^59,60^ Total energy differences between low-spin (*S* = 1/2) and high-spin (*S* = 3/2) configurations are reported in Figure S4.

To evaluate the expectation value of the *z*-component of the orbital angular momentum operator, ⟨*L*_*z*_⟩, for the first Kramers doublet, we begin by constructing the orbital angular momentum matrices *L*_*i*_ (*i* = *x*, *y*, *z*) in the spin-free basis. This basis is defined as the one that diagonalizes the molecular Coulomb Hamiltonian, containing only the electronic kinetic energy and Coulombic interactions. The electronic Hamiltonian *H*_el_, including spin–orbit coupling, is subsequently diagonalized in the spin-free basis to yield the spin–orbit basis, and the matrices *L*_*i*_ (*i* = *x*, *y*, *z*) are transformed into this basis. Since the matrix elements of *L*_*i*_ (*i* = *x*, *y*, *z*) depend on the molecule’s orientation in space, we rotate *L⃗* = (*L*_*x*_, *L*_*y*_, *L*_*z*_) to align its *z*-component with the easy axis of magnetization, defined by the principal eigenvector of the matrix *gg*^T^, where *g* is the electronic *g*-tensor matrix and *g*^T^ its transpose. Due to the degeneracy of the Kramers doublets and its relative phase problem, a small magnetic field of 0.1 T is applied along the easy axis of magnetization to *H*_el_ before diagonalization. Finally, the expectation value ⟨*L*_*z*_⟩ is obtained as the first diagonal element of the transformed *L*_*z*_ matrix.

### Assignment of Reference Polyhedron

To connect a given geometry with a reference polyhedron, we start by analyzing the number and composition of the first coordination sphere. This involves examining the distribution of distances between the Co ion and all other elements in the compounds, as shown in Figure S5. For each element *i*, we identify the position of the first and the second peak in this distribution as *d*_1,*i*_ and *d*_2,*i*_, respectively. These values are reported in Table S1. To determine if a specific atom of species *i* is part of the first coordination sphere in a given geometry, we check if its distance from the central ion falls within the interval [0, *d*_*i*_ + λ]. For each element *i*, the value of λ is set as half of the distance between the first and the second peak, i.e., *d*_2,*i*_ – *d*_1,*i*_. For a few structures, λ has been manually adjusted following a visual inspection of the atom positions to ensure an accurate representation of the first coordination sphere.

Once the number of coordinated atoms and their positions have been determined, we use the software SHAPE^33,34^ to extract the continuous shape measure (CShM).^61^ CShM provides a metric for evaluating the distance between the input geometry and a reference polyhedron model. For compounds with coordination number 4, CShM is computed with respect to tetrahedral, square, and seesaw polyhedra. For compounds with coordination number 6, the hexagonal, pentagonal pyramidal, octahedral, and trigonal prismatic polyhedra are considered. For each compound, the smallest value of the computed CShM is used to associate the complex with a reference polyhedron. For a few compounds, coordination numbers other than 4 and 6 were detected. After manually inspecting these cases, we excluded them from the present analysis.

## Data Availability

The databases COMPASS_set1, COMPASS_set2, COMPASS_lig1, and COMPASS_lig2 are available at DOI: 10.5281/zenodo.13712318.

## Supporting Information Available

Additional computational results and description of the COMPASS database (PDF)
