# Supplementary material for: Charting Regions of Cobalt’s Chemical Space with Maximally Large Magnetic Anisotropy: A Computational High-Throughput Study
Source: J Am Chem Soc. 2024 Nov 27;146(49):34158–66. doi: 10.1021/jacs.4c14076 (PMC11638940; doi:10.1021/jacs.4c14076)
Supplement: Supplementary file 1 — ja4c14076_si_001.pdf [file ja4c14076_si_001.pdf]

Supplementary Information: Charting regions of Cobalt's chemical space with maximally large magnetic anisotropy: A computational high-throughput study

Lorenzo A. Mariano, Vu Ha Anh Nguyen, Valerio Briganti, and Alessandro Lunghi*

School of Physics, AMBER and CRANN Institute, Trinity College, Dublin 2, Ireland

* lunghia@tcd.ie

DATABASE DESCRIPTION

The database COMPASS (CObalt-based Magnetic Properties from Ab initio Structural-Study), available at DOI:10.5281/zenodo.13712318, contains information on **Set-1** (COMPASS_set1), **Set-2** (COMPASS_set2), the ligands used to construct the studied compounds (COMPASS_lig1), and the totality of the ligands extracted from the CSD database (COMPASS_lig2). For **Set-1** and **Set-2**, all relevant information is summarized in two text files: COMPASS_set1.txt and COMPASS_set2.txt, respectively. Each file provides the following details for each compound: ID, anisotropy D, rhombic parameter E, $\langle L_z \rangle$, coordination number, elements in the first coordination sphere, and the computed Continuous Shape Measures (CShM) for the reference coordination, ordered as in the standard SHAPE output. Each compound ID corresponds to an ID.out and ID.xyz file stored in COMPASS_lig1 and COMPASS_lig2. The ID.out file contains the results of the CASSCF calculation, while the ID.xyz file includes the DFT-optimized structure.
For the ligands used for the construction of **Set-1** and **Set-2**, information is summarized in the COMPASS_lig1.txt file, which includes the following details: ID, CSD identifier of the compounds from which the ligand was extracted, binding atom, and the total computed charge. Each entry also has its corresponding structure saved in the ID.xyz file in COMPASS_lig1. In each ligand xyz file, the binding atom and its position are specified on the second line.

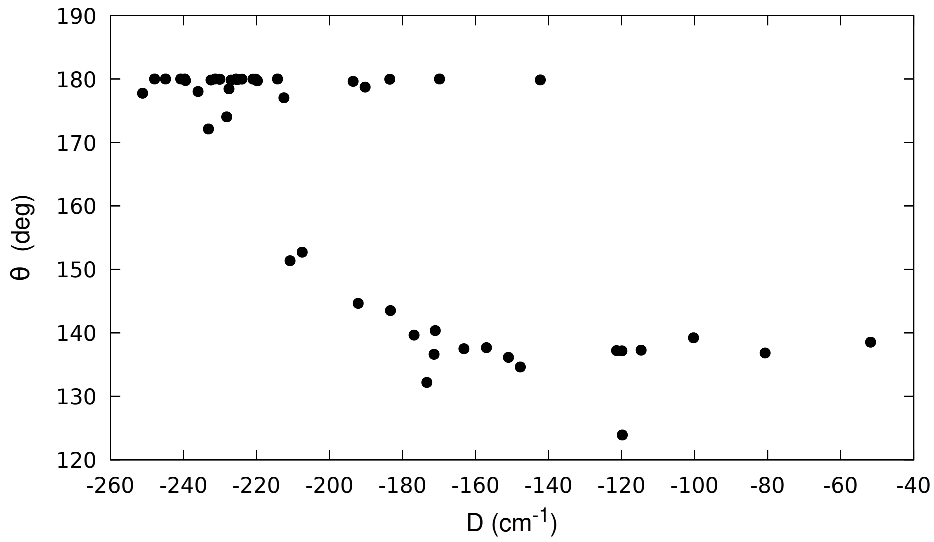

FIG. S1: Angle θ in degrees between the Co ion and the two coordinated ligands for complexes in **Set-2**.

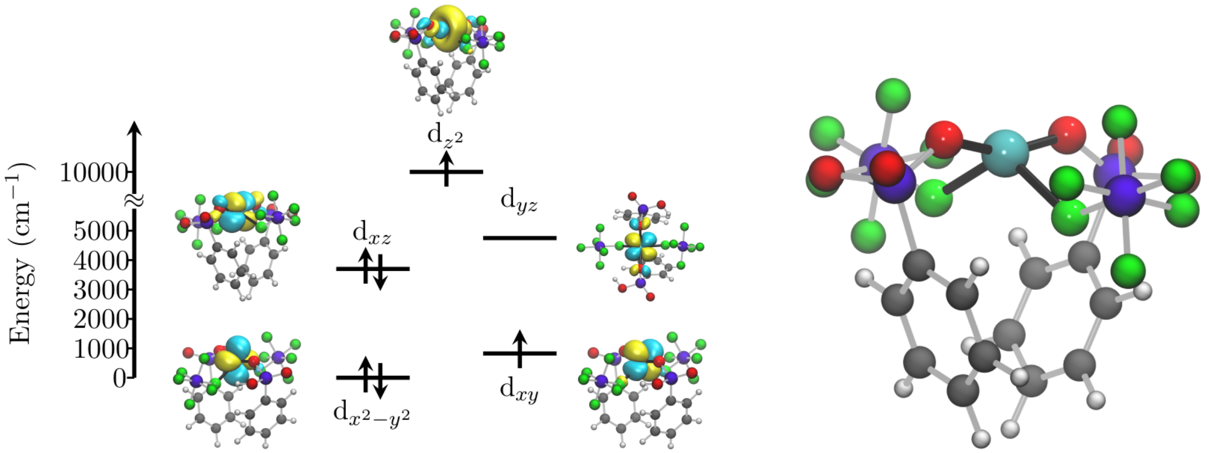

FIG. S2: MOs diagram, orbitals occupation, and optimised DFT geometry for the best 4-coordinated compound obtained in this study. CASSCF orbitals are shown next to each 3d electronic level. Color code: cyan for Co, red for O, grey for C, white for H, green for F, and purple for P.

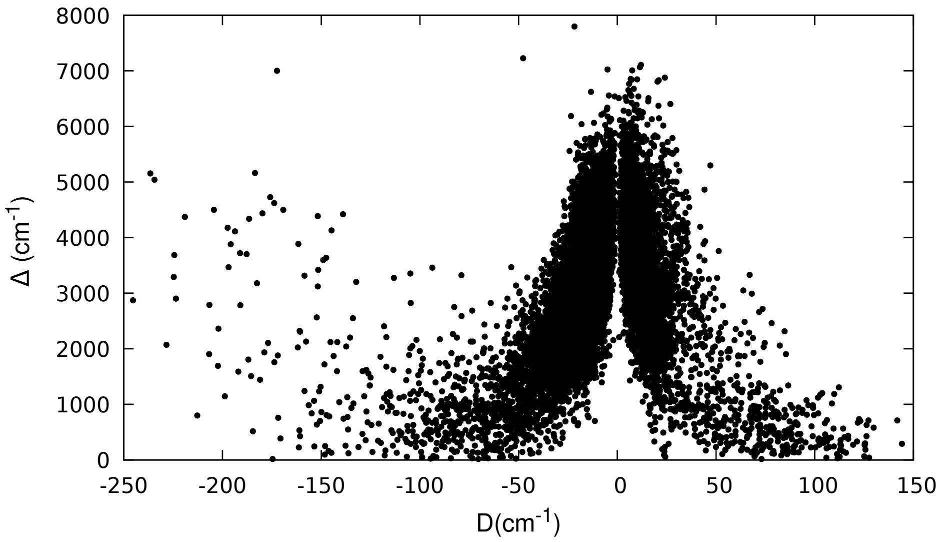

FIG. S3: Energy difference between the second and the third eigenvalues of the Ab Initio Ligand Field Theory (AILFT) Hamiltonian for each compound in **Set-1**. All values are in cm^{-1}

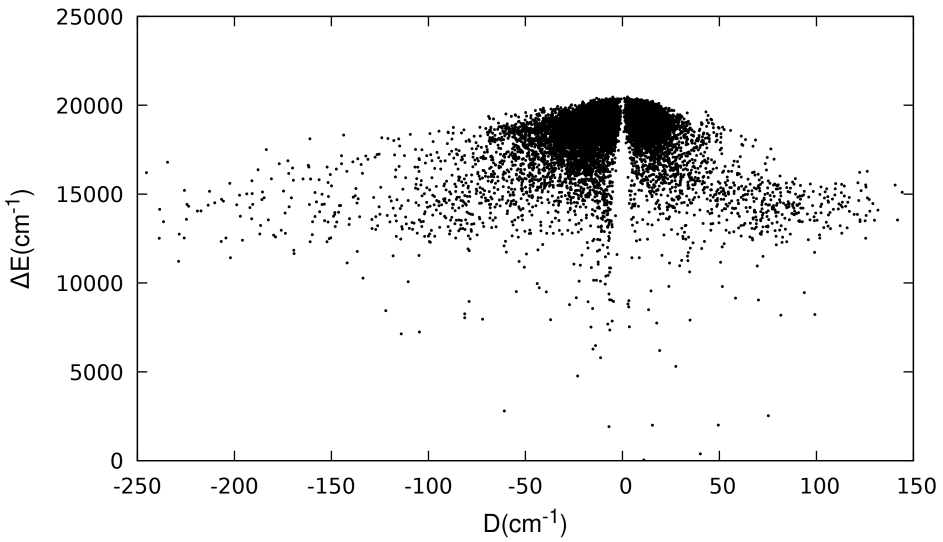

FIG. S4: The energy difference (ΔE) in cm^{-1} between the low-spin and high-spin states is calculated as the difference between the lowest $S = 1/2$ CASSCF solution and the lowest $S = 3/2$ CASSCF solution.

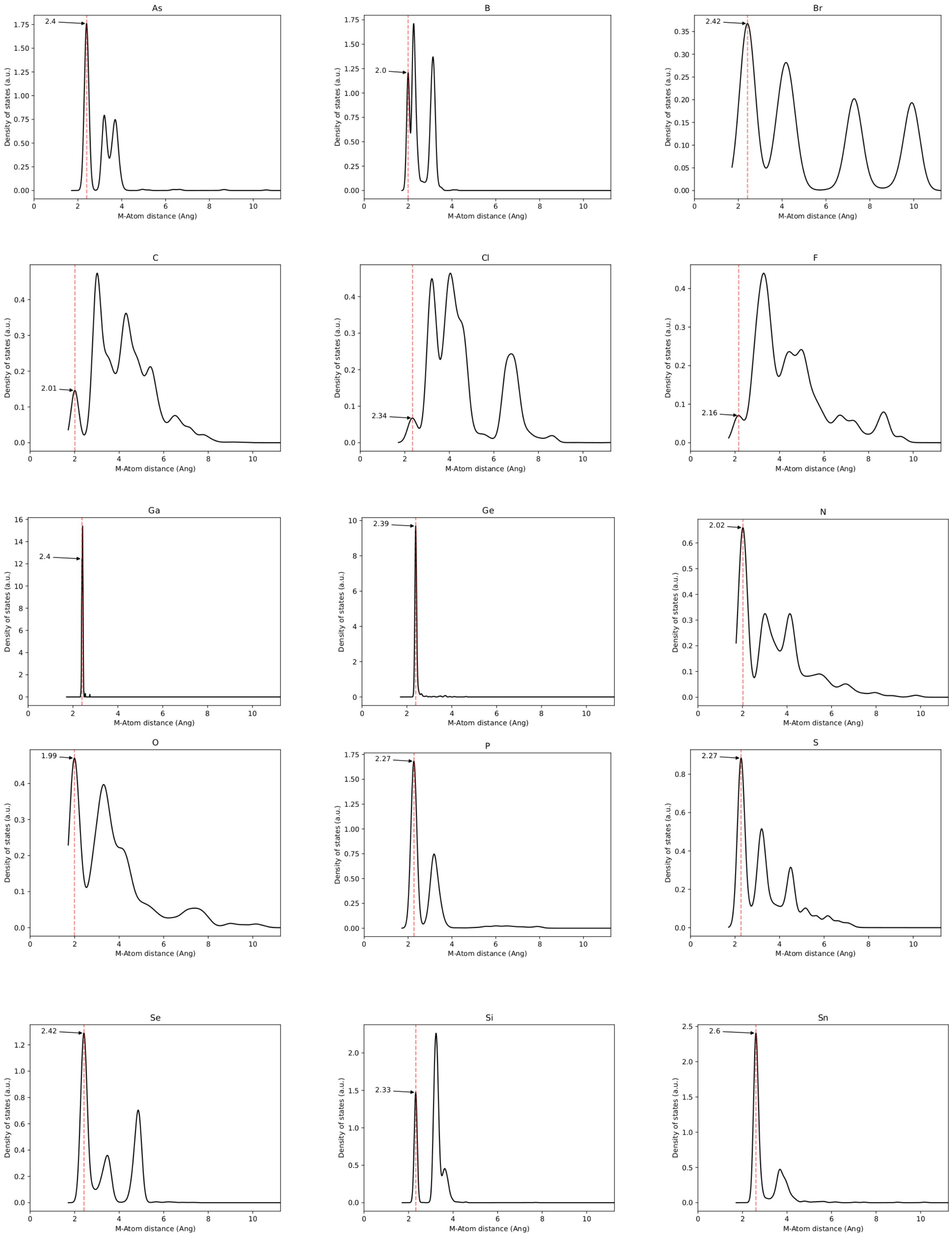

FIG. S5: Distribution of distances between the Co ion and each other element in the set. A red dashed line indicates the position of the first peak in the distribution. These values are also reported in Table S1.

Element	d_1 (Ang)	d_2 (Ang)
As	2.401	3.203
B	2.001	2.258
Br	2.421	4.177
C	2.01	3.012
Cl	2.344	3.213
F	2.163	3.289
Ga	2.401	2.43
Ge	2.392	2.64
N	2.019	3.012
O	1.991	3.308
P	2.268	3.184
S	2.268	3.194
Se	2.421	3.471
Si	2.325	2.621
Sn	2.602	3.681

TABLE S1: Position of the first (d_1) and second (d_2) peak in the Co-atom distributions reported in Fig. S5.

	AMD EPYC 7H12	Intel Xeon Gold 6148	Average
Carbon footprint (T CO_2e)	1.62	9.15	5.385
Energy needed (MWh)	15.97	27.22	21.595

TABLE S2: Estimation of the carbon footprint in tonnes of carbon dioxide equivalent (T CO_2e) and energy consumption (MWh) for the calculations performed. Two clusters were used: one with AMD EPYC 7H12 processors located in Luxembourg, and another with Intel Xeon Gold 6148 processors located in Ireland. We estimated a total of 100 CPU hours per compound, with a total of 21,528 four-coordinated compounds and 208 linear compounds, resulting in 21,736 compounds overall. Since the calculations were equally distributed across both machines, the reported average consumption between the two provides the best estimate of the total energy usage associated with this work. This estimation has been performed with the Green Algorithms tool (http://calculator.green-algorithms.org/).
